# Preoperative Atrial Fibrillation Does Not Impact Long‐Term Survival and Complications in Left Ventricular Assistance Device Recipients

**DOI:** 10.1111/aor.70020

**Published:** 2025-10-04

**Authors:** Miloud Cherbi, Vincent Galand, Valentin Barré, Pierre Groussin, Melvyn Dezecot, Paul Gautier, Philippe Maury, Clément Delmas, Erwan Flecher, Raphael Martins

**Affiliations:** ^1^ Cardiology Department Toulouse University Hospital Toulouse France; ^2^ Interventional Cardiology Department Saint Joseph Clinic Trélazé France; ^3^ Univ Rennes, CHU Rennes CIC 1414, INSERM, LTSI ‐ UMR 1099 Rennes France

**Keywords:** atrial fibrillation, heartmate, heartware, left ventricular assist device, stroke, ventricular arrhythmia

## Abstract

**Introduction:**

A substantial proportion of patients receiving left ventricular assist devices (LVADs) present with pre‐existing atrial fibrillation (AF). However, the prognostic significance of AF—particularly regarding overall survival and ventricular arrhythmias (VAs)—remains unclear.

**Methods:**

Patients included were those from the multicenter ASSIST‐ICD observational study. The association between AF and the primary endpoint of all‐cause mortality was evaluated using a 1:1 propensity score‐matched cohort. Secondary outcomes included cardiovascular and non‐cardiac mortality, bleeding, stroke, pump thrombosis, and the occurrence of early (≤ 30 days post‐implant) and late VAs.

**Results:**

Among 652 LVAD recipients, 286 patients (43.9%) had a history of AF before LVAD implantation, with a median follow‐up of 9.1 months (2.5–22.1). AF patients were older, with higher rates of dilated cardiomyopathy, a history of VAs, and longer heart failure duration. After matching, AF was not associated with higher mortality (HR 0.93 [0.69–1.26]). AF subtype (paroxysmal, persistent, permanent) had no impact on mortality. There were no significant differences in cardiovascular/non‐cardiac mortality, bleeding, ischemic stroke, pump thrombosis, or early VAs. However, AF was linked to a higher incidence of late VAs.

**Conclusion:**

In this large multicenter study, AF before LVAD implantation was not associated with increased risks of mortality, bleeding, stroke, or pump thrombosis, but was linked to a higher incidence of late VAs. These findings, based on earlier‐generation devices, should be interpreted cautiously given the recent adoption of the HeartMate 3, offering improved hemocompatibility. Further studies are needed to identify LVAD patients where AF carries clinical significance and guide optimal management.

## Graphical abstract

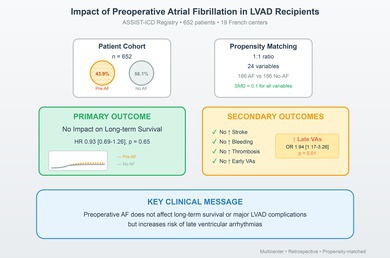

**Abbreviations**
ACE‐I: angiotensin‐converting enzyme inhibitor
AF: atrial fibrillation
aMCS: acute mechanical circulatory support
ARB: angiotensin receptor blocker
BMI: body mass index
BTT: bridge‐to‐transplant
CI: confidence interval
DT: destination therapy
HF: heart failure
HR: hazard ratio
ICD: implantable cardioverter‐defibrillator
INR: international normalized ratio
IQR: interquartile range
LVAD: left ventricular assist device
LVEDD: left ventricular end‐diastolic diameter
LVEF: left ventricular ejection fraction
MRA: mineralocorticoid receptor antagonist
OR: odds ratio
pre‐AF: preoperative atrial fibrillation
VA: ventricular arrythmia

## 1 Introduction

Given the rising prevalence of advanced heart failure (HF), left ventricular assist devices (LVADs) have become a cornerstone of contemporary care, widely adopted to enhance both longevity and quality of life, whether as a bridge to transplant (BTT) or a destination therapy (DT) option [1].

Atrial fibrillation (AF) is common in patients with end‐stage HF and contributes to increased morbidity and mortality, largely due to its detrimental effect on cardiac output [2, 3]. However, its prognostic relevance in the context of LVAD implantation remains uncertain, as the device provides continuous and robust hemodynamic support. While some studies have reported an association between preoperative AF (pre‐AF) and worse outcomes, including higher mortality and HF readmissions [4, 5, 6], others have not confirmed these observations [6, 7]. Similarly, evidence regarding the relationship between pre‐AF and LVAD‐related complications remains inconsistent across studies.

To clarify those controversial issues, we studied the clinical impact of pre‐AF in post‐operative outcomes, in patients implanted with a LVAD in the nationwide ASSIST‐ICD registry.

## 2 Methods

### 2.1 Study Design

The ASSIST‐ICD (Determination of Risk Factors of Ventricular Arrhythmias After Implantation of Continuous Flow Left Ventricular Assist Device with Continuous Flow Left Ventricular Assist Device) study is a retrospective, multicenter observational study (NCT02873169) of durable mechanical circulatory support devices implanted in 19 tertiary French centers. The study methods have been previously reported [8]. Briefly, patients > 18 years of age who had been implanted with axial HeartMate II (Abbott, Chicago, Illinois), Jarvik 2000 (Jarvik Heart, New York, New York), or centrifugal HeartWare pumps (Medtronic, Columbia Heights, Minnesota) between February 2006 and December 2016 were included. The type of pump implanted depended on the local heart team's decision in each center. Exclusion criteria were patients who underwent total artificial heart placement or pulsatile‐flow LVAD, a history of heart transplant, and VentrAssist (Ventracor, Chatswood, Australia) recipients.

This study was approved by the regional ethic committees, the French Advisory Committee on the Treatment of Research Information in the Field of Health, and the French National Commission of Informatics and Civil Liberties.

### 2.2 Baseline Assessment and Follow‐Up

Baseline data—including demographic characteristics (age, sex, body mass index [BMI]), cardiac disease and HF history, history of ventricular arrhythmias (VAs) before LVAD implantation, HF medical therapy (beta‐blocker, angiotensin‐converting enzyme inhibitor [ACE‐I], angiotensin receptor blocker [ARB], mineralocorticoid receptor antagonist [MRA]), previous implantable cardioverter‐defibrillator (ICD), echocardiography (left ventricular ejection fraction [LVEF], left ventricular end‐diastolic diameter [LVEDD]), and blood chemistry values—were collected from hospital files for all enrolled patients. The echocardiographic and blood sample data used for the analysis were the last performed before LVAD implantation. Perioperative data were also collected, including information on concomitant surgical procedures performed during LVAD implantation and the need for acute mechanical circulatory support (aMCS) before or after surgery. Follow‐up was performed according to each institution's protocols.

### 2.3 Atrial Fibrillation

In this study, AF was diagnosed by ECG documentation and categorized as paroxysmal, persistent/long‐standing persistent, or permanent AF using the standard definitions [9]: (1) Paroxysmal AF was defined in case of self‐terminating or medically reduced AF; (2) persistent AF was defined if an electrical shock was necessary, and AF was categorized as “long standing” if AF lasted for > 1 year but with a strategy of rhythm control; (3) AF was defined as permanent if the arrhythmia was accepted, indicating that no further attempts at restoration of sinus rhythm were planned. Of note, AF was classified as paroxysmal, persistent/long‐standing persistent, or permanent depending on the arrhythmia pattern at the time of the LVAD implantation.

### 2.4 Study Endpoints

The primary endpoint of the study was all‐cause mortality in LVAD patients with versus without pre‐AF. Secondary endpoints included cardiovascular mortality (of cardiac or vascular origin), non‐cardiovascular mortality, early and late VAs, early electrical storm, and LVAD‐related complications such as thrombosis, stroke, and bleeding. Early VAs/electrical storm were defined as occurring within 30 days of LVAD implantation, while late VAs occurred beyond this period.

### 2.5 Statistical Analysis

Continuous variables are presented as medians with interquartile ranges (IQR), and categorical variables as counts with corresponding percentages. Comparisons between groups were performed using the Mann–Whitney *U* test for continuous variables, and either the chi‐square or Fisher's exact test for categorical variables, as appropriate. All‐cause, cardiovascular, and non‐cardiovascular mortality were analyzed using Kaplan–Meier time‐to‐event curves to compare outcomes between LVAD patients with and without pre‐AF. Survival distributions were compared with the log‐rank test, and hazard ratios (HRs) with 95% confidence intervals (CIs) were estimated using Cox proportional hazards regression. The occurrence of early and late VAs was assessed using logistic regression, with results expressed as odds ratios (ORs) and 95% CIs.

To account for potential confounding factors, propensity score‐matched cohorts were constructed based on the following covariates: age, sex, BMI, cardiovascular risk factors (diabetes mellitus, hypertension, dyslipidemia, smoking), history of VAs, previous ICD, type of cardiomyopathy, HF duration, LVEF, and LVEDD prior to LVAD implantation, LVAD type and indication (BTT or DT), pre‐implantation creatinine, bilirubin, and sodium levels, presence of cardiogenic shock or acute aMCS before LVAD implantation, combined surgery with LVAD, and the use of beta‐blockers, ACE‐Is, ARBs, and MRAs before LVAD implantation. Missing data were addressed using multiple imputations with chained equations, performed with the R package *mice*, generating 10 imputed datasets. In these datasets, propensity scores for pre‐AF were calculated using a logistic regression model and subsequently averaged. Using these propensity scores, patients with pre‐AF were matched in a 1:1 ratio to patients without pre‐AF through the nearest neighbor method, applying a caliper of 0.1 and without replacement. The balance of potential confounders between the study groups was assessed using the standardized mean difference (SMD), with a value below 0.10 indicating no significant difference, and results were presented as Love plots. Additionally, subgroup analyses were performed to assess whether the impact of pre‐AF varied by age, sex, type of cardiomyopathy (ischemic vs. non‐ischemic), LVEF (≤ 20% vs. > 20%), LVAD type (Heartmate 2, Heartware), and LVAD indication (BTT vs. DT). Finally, a subgroup analysis was conducted to compare the different subtypes of preoperative AF (paroxysmal, persistent, and permanent) both with each other and with patients without pre‐AF.

All tests were two‐tailed. A value of *p* ≤ 0.05 was accepted as statistically significant. Analyses were performed using R software [version 4.3.2 (2023‐10‐31)].

## 3 Results

### 3.1 Unmatched Cohort

**FIGURE 1:**
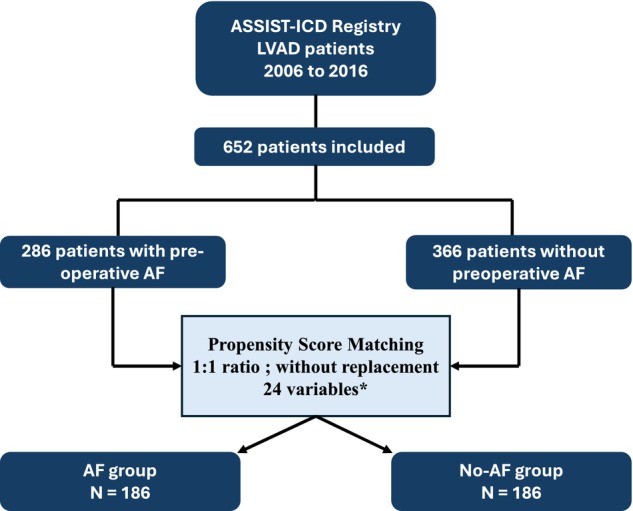
Flow chart of the study. AF, atrial fibrillation; LVAD, left ventricular assist device. *Covariates used for propensity‐score matching: Age, sex, BMI, cardiovascular risk factors (diabetes mellitus, hypertension, dyslipidemia, smoking), history of VAs, previous ICD, type of cardiomyopathy, HF duration, LVEF and LVEDD prior to LVAD implantation, LVAD type and indication (BTT or DT), pre‐implantation creatinine, bilirubin and sodium levels, presence of cardiogenic shock or acute aMCS before LVAD implantation, combined surgery with LVAD, and the use of beta‐blockers, ACE‐Is, ARBs, and MRAs before LVAD implantation. [Color figure can be viewed at wileyonlinelibrary.com]

From 2006 to 2016, a total of 652 patients were included in the final analysis and followed during 9.1 (2.5–22.1) months (Figure 1). Among the overall population, 286 (43.9%) and 16 (2.5%) had a history of AF or flutter/atrial tachycardia (1 atrial tachycardia and 15 flutter), respectively. The clinical characteristics of these 286 AF patients are summarized in Table 1. Briefly, patients with a history of AF were significantly older (64.2 vs. 58.2 years, *p* < 0.01) and most likely men (89.9% vs. 83.1%, *p* = 0.02). There was no difference regarding cardiovascular risk factors except a lower rate of smokers among patients with AF. Interestingly, patients with AF were more likely to have dilated cardiomyopathies (35.3% vs. 21.0%; *p* < 0.01) with a longer HF duration (113.0 vs. 12.3 months, *p* < 0.01) and more dilated left ventricles. They also more often had a history of VAs prior to LVAD (42.0% vs. 28.1%, *p* < 0.01) and were more frequently implanted with ICD (73.1% vs. 53.0%, *p* < 0.01). Additionally, beta‐blockers and MRAs were more frequently prescribed prior to LVAD in pre‐AF patients. Lastly, although the type of LVAD implanted did not differ between groups, patients with AF were significantly more likely to receive the device as DT (46.5% vs. 31.1%, *p* < 0.01) and had a lower prevalence of pre‐implant aMCS use (20.6% vs. 35.2%, *p* < 0.01).

**TABLE 1:** Baseline characteristics.

	Unmatched cohort				Matched cohort		
	AF (*n* = 286)	No‐AF (*n* = 366)	Missing values	*p*	AF (*n* = 186)	No‐AF (*n* = 186)	SMD
Age, median (IQR), years	64.2 (56.3–69.1)	58.2 (49.8–65.4)	0.0%	< 0.01	62.3 (54.8–67.8)	61.3 (54.6–67.5)	0.06
Male sex, *n* (%)	257 (89.9)	304 (83.1)	0.0%	0.02	164 (88.2)	164 (88.2)	0.00
BMI ≥ 30 kg/m^2^	53 (18.5)	51 (13.9)	0.0%	0.14	30 (16.1)	31 (16.7)	−0.01
Diabetes mellitus, *n* (%)	74 (25.9)	80 (21.9)	0.0%	0.27	41 (22.0)	42 (22.6)	−0.01
Hypertension, *n* (%)	111 (38.8)	122 (33.3)	0.0%	0.17	68 (36.6)	66 (35.5)	0.02
Dyslipidemia, *n* (%)	119 (41.8) (*n* = 285)	164 (44.8)	0.2%	0.48	80 (43.0)	74 (39.8)	0.07
Smoking, *n* (%)	155 (54.2)	240 (65.6)	0.0%	< 0.01	105 (56.5)	108 (58.1)	−0.03
History of VA, *n* (%)	120 (42.0)	103 (28.1)	0.0%	< 0.01	73 (39.2)	75 (40.3)	−0.02
Previous ICD, *n* (%)	209 (73.1)	194 (53.0)	0.0	< 0.01	127 (68.3)	128 (68.8)	−0.01
Heart failure etiology, *n* (%)							
Ischemic	156 (54.5)	256 (69.9)	0.0%	< 0.01	106 (57.0)	115 (61.8)	0.02
Dilated	101 (35.3)	77 (21.0)			66 (35.5)	51 (27.4)	
Other	29 (10.1)	33 (9.0)			14 (7.5)	20 (10.8)	
Heart failure duration, median (IQR), months	113.0 (41.7–207.6)	12.3 (0.9–111.8)	0.0%	< 0.01	106.3 (22.2–189.8)	80.8 (2.1–174.7)	0.07
Echocardiographic data before LVAD implantation, median (IQR)							
LVEF, %	20.0 (15.0–25.0) (*n* = 280)	20.0 (15.0–25.0)	3.7%	0.62	20.0 (15.0–25.0)	20.0 (15.0–25.0)	0.02
LVEDD, mm	71.0 (64.0–77.0) (*n* = 259)	69.0 (63.0–73.0) (*n* = 315)	12.0%	< 0.01	71.0 (64.3–77.0)	69.5 (65.0–75.0)	0.07
Laboratory values, median (IQR)							
Serum creatinine level, μmol/L	126.0 (96.3–155.8) (*n* = 266)	107.5 (81.0–141.3) (*n* = 332)	8.3%	< 0.01	117.5 (91.0–152.8)	117.5 (88.3–145.0)	0.07
Serum sodium level, mmol/L	135.0 (132.0–138.0) (*n* = 263)	136.0 (132.5–139.0) (*n* = 331)	8.9%	0.04	136.0 (132.0–139.0)	136.0 (132.0–139.0)	−0.06
Total bilirubin level, μmol/L	16.0 (11.0–27.0) (*n* = 249)	16.0 (10.0–27.0) (*n* = 314)	13.7%	0.52	15.0 (10.0–25.8)	16.0 (10.0–27.0)	−0.05
Type of LVAD, *n* (%)							
Heartmate II	203 (71.0)	272 (74.3)	0.0%	0.63	130 (70.0)	137 (73.7)	0.06
HeartWare	59 (20.6)	68 (18.6)			41 (22.0)	34 (18.3)	
Jarvik 2000	24 (8.4)	26 (7.1)			15 (8.1)	15 (8.1)	
LVAD indication, *n* (%)							
Bridge to transplantation	148 (51.7)	239 (65.3)	0.0%	< 0.01	101 (54.3)	107 (57.5)	0.03
Destination therapy	133 (46.5)	114 (31.1)			82 (44.1)	73 (39.2)	
Bridge to decision/recovery	5 (1.7)	13 (3.6)			3 (1.6)	6 (3.2)	
Operative characteristics, *n* (%)							
Cardiogenic shock before LVAD implantation	146 (51.0)	202 (55.2)	0.0%	0.33	90 (48.4)	99 (53.2)	−0.10
Acute MCS before LVAD implantation	59 (20.6)	129 (35.2)	0.0%	< 0.01	44 (23.7)	52 (28.0)	−0.10
ECMO	38 (13.3)	98 (26.8)			31 (16.7)	38 (20.4)	
Impella	21 (7.3)	43 (11.7)			15 (8.1)	16 (8.6)	
IABP	15 (5.2)	43 (11.7)			11 (5.9)	17 (9.1)	
Combined surgery with LVAD	48 (18.0)	47 (12.8)	0.0%	0.19	27 (14.5)	20 (10.8)	0.04
Treatment before LVAD implantation, *n* (%)							
Betablocker	210 (73.4)	213 (58.2)	0.0%	< 0.01	134 (72.0)	130 (69.9)	0.05
ACE‐I	167 (58.6) (*n* = 285)	185 (51.8) (*n* = 357)	1.5%	0.10	109 (58.6)	107 (57.5)	0.02
ARB	40 (15.0) (*n* = 266)	28 (8.5) (*n* = 330)	8.6%	0.02	21 (11.3)	21 (11.3)	0.00
MRA	179 (62.8) (*n* = 285)	177 (49.7) (*n* = 356)	1.7%	< 0.01	117 (62.9)	110 (59.1)	0.08
Amiodarone	172 (93.5) (*n* = 284)	119 (33.4) (*n* = 356)	1.8%	< 0.01	95 (51.1)	98 (52.7)	−0.03

Abbreviations: ACE‐I, angiotensin‐converting enzyme; AF, atrial fibrillation; ARB, Angiotensin receptor blockers; BMI, body mass index; IQR, interquartile; LVAD, left ventricular assist device; LVEDD, left ventricular end‐diastolic diameter; LVEF, left ventricular ejection fraction; MRA, mineralocorticoid receptor antagonist; SMD, standardized mean difference; VA, ventricular arrhythmia.

### 3.2 Matched Cohort

Propensity score matching without replacement yielded a final cohort of 372 patients, with 186 in the AF group, achieving a 1:1 matching ratio. The median duration of follow‐up was comparable to that of the unmatched cohort, at 9.2 (2.4–21.6) months. As shown in the Love plot (Figure S1), all covariates used in the matching process were well balanced between groups, with SMDs below 0.1, indicating minimal residual bias. Additionally, as presented in Table 1, the matching process also effectively balanced baseline characteristics that were not explicitly included in the propensity score model.

### 3.3 Outcomes

**FIGURE 2:**
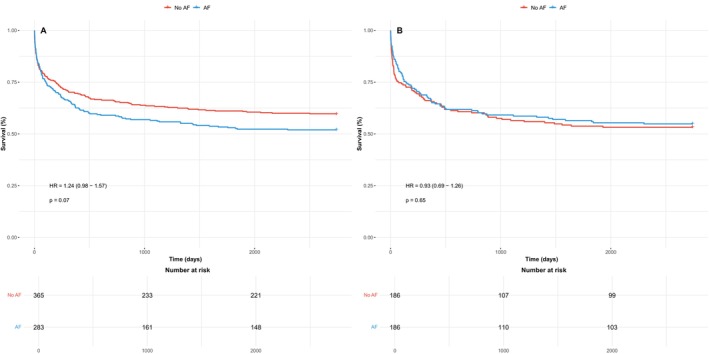
Kaplan–Meier curves for all‐cause mortality in the unmatched (A) and matched (B) study cohort comparing LVAD patients with and without preoperative AF. AF, atrial fibrillation; HR, hazard ratio; LVAD, left ventricular assist device. [Color figure can be viewed at wileyonlinelibrary.com]

**FIGURE 3:**
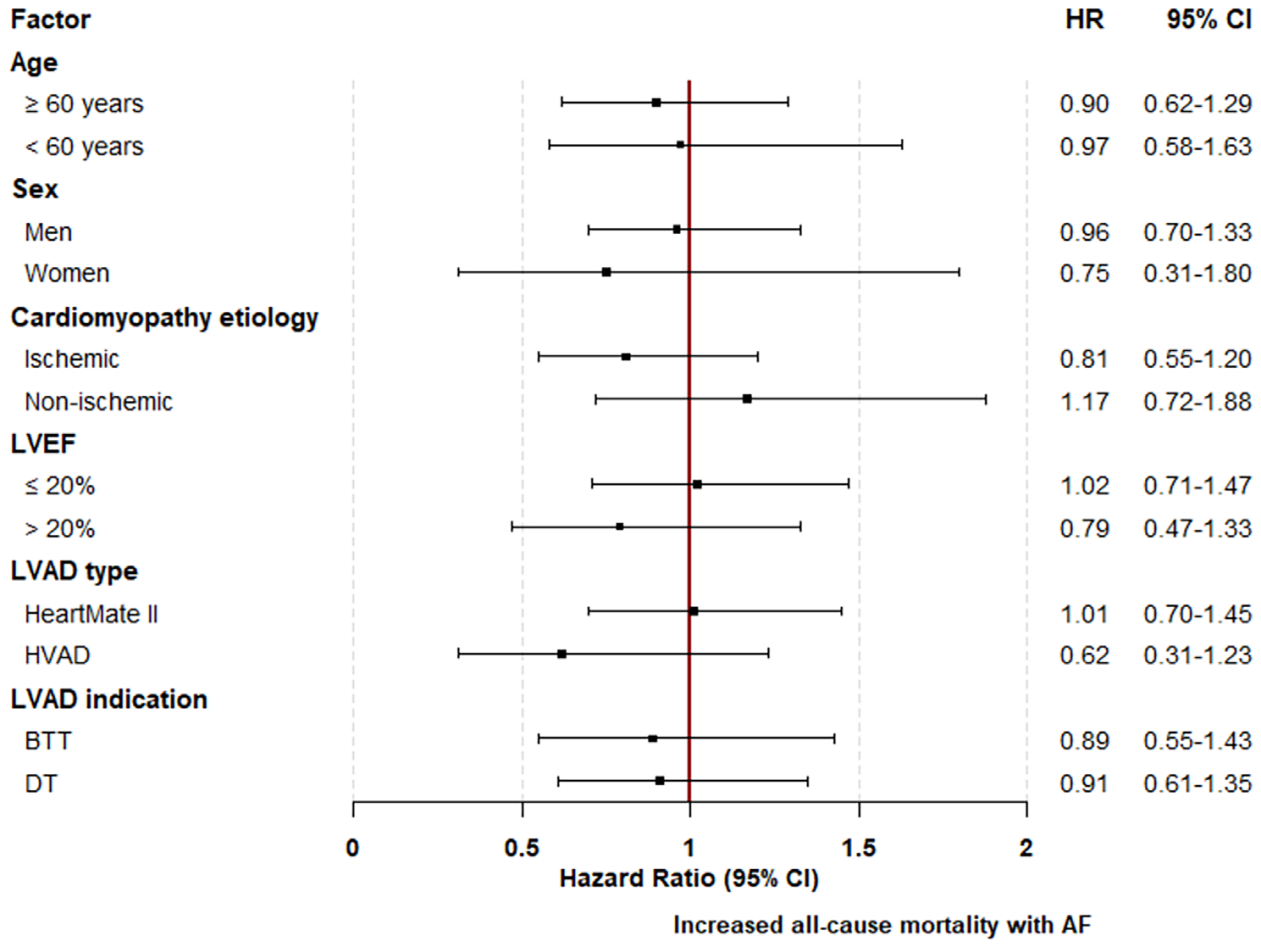
Association between preoperative AF and all‐cause mortality in subgroups of interest from the matched study cohort of LVAD patients. AF, atrial fibrillation; BTT, bridge to transplant; CI, confidence interval; DT, destination therapy; HR, hazard ratio; LVAD, left ventricular assist device; LVEF, left ventricular ejection fraction. [Color figure can be viewed at wileyonlinelibrary.com]

**FIGURE 4:**
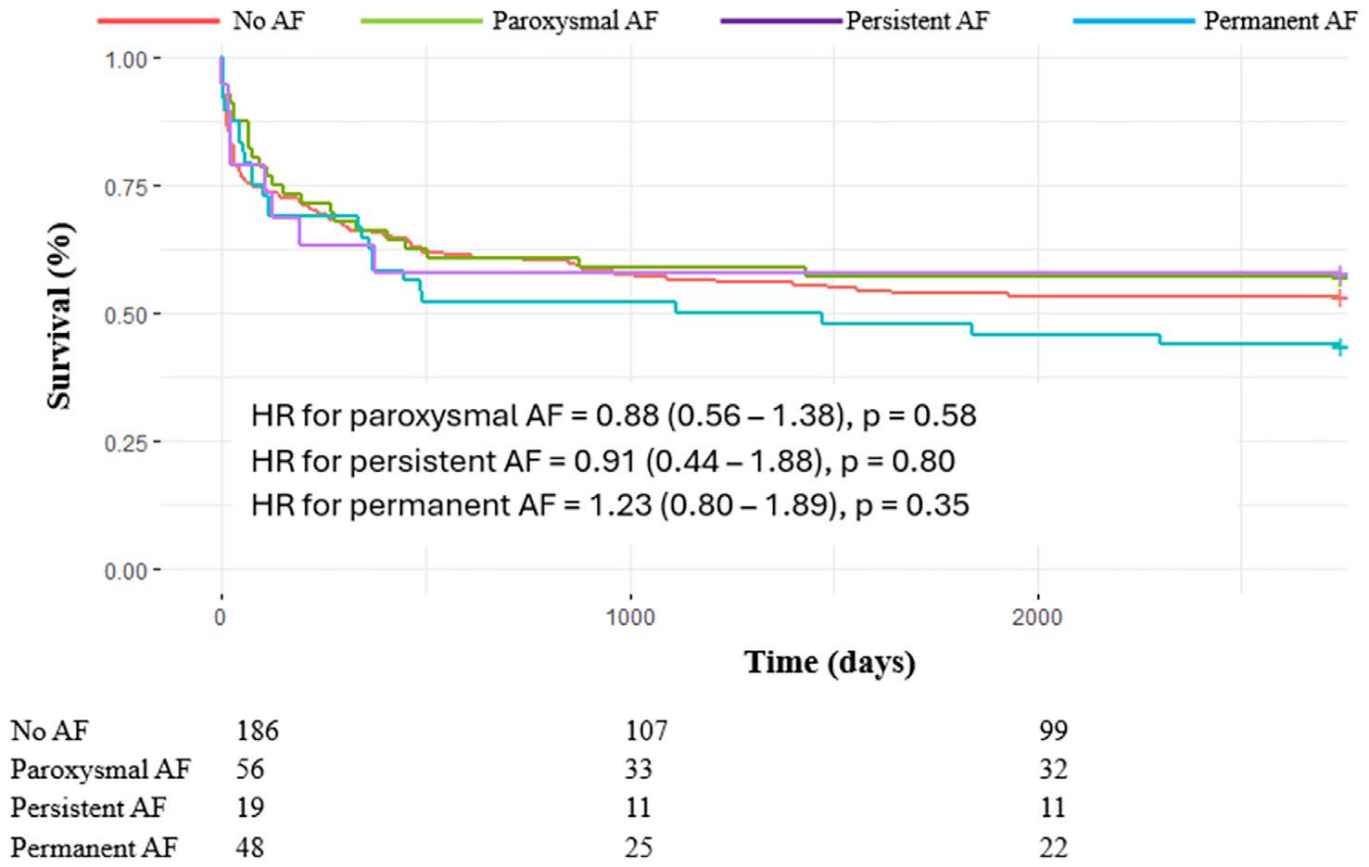
Kaplan–Meier curves for all‐cause mortality in the matched cohort stratified by subtype of preoperative AF. AF, atrial fibrillation; HR, hazard ratio; LVAD, left ventricular assist device. [Color figure can be viewed at wileyonlinelibrary.com]

As shown in Figure 2, a history of AF prior to LVAD implantation had no significant impact on long‐term survival, both in the unmatched cohort (HR 1.24 [0.98–1.57], *p* = 0.07) and in the matched cohort (HR 0.93 [0.69–1.26], *p* = 0.65). This neutral effect of pre‐AF on all‐cause mortality was consistent across all predefined subgroups, including age, sex, type of cardiomyopathy (ischemic vs. non‐ischemic), LVAD indication (bridge to transplant vs. destination therapy), heart failure duration (≥ 1 year vs. <1 year), and baseline LVEF (≤ 20% vs. > 20%) (Figure 3). Furthermore, the AF subtype (paroxysmal, persistent, or permanent) had no impact on mortality when compared to patients without pre‐AF (Figure 4).

Pre‐AF was also not associated with increased risk of cardiovascular death (HR 1.11 [0.72–1.70], *p* = 0.64), non‐cardiovascular death (HR 0.82 [0.53–1.25], *p* = 0.35), early VAs (OR 0.97 [0.62–1.53], *p* = 0.91), early electrical storms (OR 0.67 [0.29–1.47], *p* = 0.32), bleeding (OR 0.81 [0.48–1.36], *p* = 0.43), ischemic stroke (OR 0.86 [0.47–1.59], *p* = 0.64), or pump thrombosis (OR 1.11 [0.59–2.09], *p* = 0.75) (Table 2). However, pre‐AF was significantly associated with a higher incidence of late VAs (OR 1.94 [1.17–3.26], *p* = 0.01).

**TABLE 2:** Secondary outcomes in LVAD patients with and without pre‐AF in the matched cohort.

	HR or OR^a^ (95% CI)	*p*
Cardiovascular death	1.11 (0.72–1.70)	0.64
Non‐cardiac death	0.82 (0.53–1.25)	0.35
Early VAs (≤ 30 days)	0.97 (0.62–1.53)	0.91
Late VAs (> 30 days)	1.94 (1.17–3.26)	0.01
Early electrical storm (≤ 30 days)	0.67 (0.29–1.47)	0.32
Bleeding	0.81 (0.48–1.36)	0.43
Ischemic stroke	0.86 (0.47–1.59)	0.64
Pimp thrombosis	1.11 (0.59–2.09)	0.75

Abbreviations: CI, confidence interval; HR, hazard ratio; OR, odds ratio; VA, ventricular arrhythmia.

^a^ Cardiovascular and non‐cardiac mortality are presented as hazard ratios with 95% confidence intervals. Early and late VAs, bleeding, stroke, and pump thrombosis are presented as odds ratios and 95% CIs.

## 4 Discussion

In this large multicenter study, we report on the prognostic significance of preoperative AF in LVAD recipients, based on data from a nationwide registry. Our main findings are as follows: (1) AF was highly prevalent at the time of LVAD implantation, affecting 43.9% of patients; (2) a history of AF did not influence long‐term survival, whether considering all‐cause, cardiovascular, or non‐cardiovascular mortality; (3) survival outcomes were not affected by the subtype of AF, including paroxysmal, persistent, or permanent forms; and (4) the presence of pre‐AF was not associated with an increased risk of LVAD‐related complications such as early VAs/electrical storms, bleeding, stroke, or pump thrombosis—although it was significantly associated with a higher incidence of late VAs.

While AF is a very common cardiac condition—particularly prevalent in patients with advanced HF and those supported with LVADs [10]—data regarding the clinical impact of preoperative AF remain limited and inconsistent, largely derived from retrospective, single‐center studies [4, 5, 6, 7]. Our findings of no increased mortality associated with preoperative AF contrast with some previous reports, though closer examination reveals important nuances in AF subtype analysis, as while paroxysmal AF was not associated with worse outcomes in LVAD patients, persistent AF was an independent predictor of decreased survival and increased HF hospitalizations [4, 6]. Conversely, both Stulak et al. [11] and the INTERMACS registry [12] reported no overall survival difference between patients with and without preoperative AF, though neither provided detailed subtype analysis, which aligns more closely with our findings. Several factors may explain these discrepancies beyond AF classification. Baseline population characteristics differed notably between studies, with our cohort having a higher prevalence of dilated cardiomyopathy in AF patients and longer HF duration, potentially representing a different phenotype than previous series. Additionally, the evolution of rhythm control strategies, particularly the increased utilization of catheter ablation procedures during our study period, may have influenced AF management and outcomes. Though these series control for confounding patient factors, it remains unclear whether persistent AF identifies patients with more advanced comorbidities or whether persistent atrial arrhythmias independently impact outcomes through mechanisms such as increased right ventricular failure or late VAs after LVAD. Whereas AF is known to adversely affect the course of native HF by eliminating atrial systole, thereby impairing left ventricular filling and diastolic function, its impact in the context of LVAD support remains less clearly defined. Several factors may explain this differential impact of pre‐AF on clinical outcomes of LVAD patients. First, the LVAD provides continuous flow that may compensate for the loss of atrial kick, reducing the hemodynamic impact of AF compared to native heart function. Additionally, the device's ability to maintain cardiac output may override the typical negative effects of AF on ventricular filling. Furthermore, left ventricular unloading from the LVAD may reduce atrial pressures and potentially improve AF management. Lastly, the intensive monitoring in LVAD patients may lead to better detection and management of AF‐related complications. Importantly, these findings must be interpreted in the context of the device era studied. All patients in our cohort were implanted between 2006 and 2016, during which the HeartMate II, HeartWare HVAD, and Jarvik 2000 were the dominant devices. This context is essential, as these older‐generation LVADs have distinct hemocompatibility profiles compared to the newer HeartMate 3, now the standard of care. Therefore, while our results reflect the clinical reality of that period, they may not be directly generalizable to the current LVAD population supported by modern devices with lower stroke and thrombosis rates.

Besides, stroke, bleeding and LVAD thrombosis are common complications in patients with LVAD, but as previously mentioned, the impact of pre‐AF on their occurrences remains unclear. First, a higher risk of stroke has been described by Stulak et al. in LVAD recipients with pre‐AF, reaching 72% after 2 years of follow‐up, compared to 46% in those patients with no history of atrial arrhythmias [11]. Other works suggested to target a higher international normalized ratio (INR) in patients with concomitant LVAD and AF [4]. Indeed, although authors found a similar bleeding or thromboembolism events rates between patients with or without AF, those with AF had a higher INR level at the time of stroke and during the 4 weeks preceding the event. However, more recent studies and meta‐analyses did not show any relationship between AF and stroke in LVAD recipients, suggesting that standard INR levels could be targeted [13]. Our study confirms these results, showing no association between AF and the rate of stroke.

In LVAD recipients, the higher risk of bleeding has been well established and is mostly due to acquired Willebrand disease [14], long‐term anticoagulation therapy, recurrent surgeries and the absence of pulse perfusion in the gastro‐intestinal tractus [15]. However, the hypothetic higher risk of bleeding among patient with pre‐AF remains controversial. Indeed, even if Xia et al. [12] found that patients with pre‐AF had a higher incidence of bleeding events compared to those without AF (0.94 and 0.77 bleeding events per patient‐year, respectively), Kurihara et al. [7] found similar bleeding episodes rate between both groups (0.19 vs. 0.22 bleeding events per patient year (*p* = 0.84)), while Enriquez et al. observed a comparable incidence of bleeding between the different subgroups of AF pattern [4].

Besides, we found no association between preoperative AF and pump thrombosis. Current evidence on this point remains conflicting [12, 16], and these results should be interpreted with caution because several unmeasured factors—most notably daytoday adherence to anticoagulation therapy, for which our registry lacks reliable data—may influence thrombosis risk. Otherwise, it is also possible that the intrinsic prothrombotic milieu created by the LVAD itself outweighs any incremental risk conferred by AF alone. As with stroke, these findings should not be extrapolated to newer‐generation devices without dedicated investigation. Given the heterogeneity of existing studies, further research is needed to identify subgroups in which AF might genuinely add to thrombotic risk, particularly in the era of the HeartMate 3, for which data remain scarce and limited to small‐sample studies that do not allow for high‐level evidence [17].

Lastly, our study demonstrated that preoperative AF was significantly associated with the occurrence of late VAs following LVAD implantation. From a mechanistic perspective, the specific association between preoperative AF and late VAs likely reflects multiple interconnected pathophysiological processes that predominantly manifest during the chronic phase following LVAD implantation. Preoperative AF is a marker of diffuse myocardial fibrosis and advanced structural remodeling affecting both atrial and ventricular tissues, creating an arrhythmogenic substrate that progressively intensifies over time [18]. This extensive myocardial involvement extends beyond isolated atrial pathology, encompassing alterations in calcium handling, gap junction function, and autonomic innervation, all of which predispose to ventricular arrhythmogenesis [19]. Importantly, this chronic substrate‐driven arrhythmogenicity fundamentally differs from the acute mechanisms underlying early VAs, which are mainly triggered by perioperative inflammatory responses, electrolyte imbalances, and surgical stress, factors that obscure the influence of pre‐existing myocardial abnormalities [20]. In contrast, as these acute factors resolve in the late phase, the long‐term electrophysiological consequences of atrial arrhythmogenesis become apparent. These include the effects of irregular RR intervals on ventricular refractoriness, progressive autonomic dysfunction linked to persistent atrial electrical instability [21], and chronic alterations in calcium handling that promote delayed afterdepolarizations. Furthermore, patients with preoperative AF may have a reduced capacity for favorable ventricular remodeling under LVAD support, leading to the formation of heterogeneous conduction zones around the device implantation site. This creates a substrate for reentrant circuits that typically become clinically manifest only after months of chronic device‐heart interaction. Additionally, underlying genetic factors may contribute to this association, as previous studies have identified common genetic variants—such as those in SCN5A and SCN10A—associated with both AF and VAs [22]. In this context, our results are consistent with previous reports [8, 23, 24], which also found a link between prior AF and late VAs, reinforcing the idea that the arrhythmic history—including both atrial and ventricular events—should be carefully considered in the pre‐implant evaluation of LVAD candidates to better stratify the risk of future VAs. The clinical translation of these findings must be considered within the broader context of late VA risk stratification in LVAD recipients. While preoperative AF represents a significant predictor, comprehensive risk assessment requires integration of multiple clinical, echocardiographic, hemodynamic, and arrhythmic parameters. Previous work has demonstrated the utility of multifactorial approaches in this population, with the VT‐LVAD score incorporating preoperative AF alongside other validated predictors to enhance risk stratification accuracy [8, 20]. Such integrated models acknowledge that late VA occurrence results from complex interactions between patient‐specific factors and post‐implantation evolution, rather than isolated risk markers. Overall, the presence of preoperative AF represents one of several factors that could inform therapeutic decisions related to rhythm management in LVAD recipients. While the benefit of ICD therapy remains uncertain in this population [25] and has been associated with increased complication rates including pocket hematomas and infections [26], enhanced risk prediction for late VAs through preoperative AF identification may contribute to better selection of patients in whom ICD therapy should be maintained, with optimized programming strategies.

Eventually, although data on catheter ablation procedures—either before or after LVAD implantation—were not available in our study, this aspect warrants further clarification. Current guidelines suggest considering ablation for highly symptomatic LVAD patients after failure of anti‐arrhythmic drugs [20], but this recommendation relies on limited evidence. In contrast, large, randomized trials such as EAST‐AFNET 4 [27] and CASTLE‐HTx [28] have demonstrated the benefits of early rhythm control, including catheter ablation, even in patients with advanced HF without LVADs. Whether such benefits extend to the LVAD population remains uncertain, as observational studies to date have not shown a clear impact on major outcomes such as mortality or hospitalizations [29]. Given the heterogeneity of AF in this setting, future research should aim to identify subgroups of LVAD patients who may be more likely to benefit from rhythm control strategies.

## 5 Limitations

Our study has several limitations inherent to its observational and retrospective design, which may have influenced the findings. Our results must be interpreted within the specific context of the studied cohort, which includes only patients implanted between 2006 and 2016 with older‐generation continuous‐flow LVADs (HeartMate II, HeartWare HVAD, Jarvik 2000). As the study period ended in 2016, it did not include patients implanted with the HeartMate 3, which has since become the standard device for new implantations due to its improved hemocompatibility profile and lower rates of pump thrombosis and stroke. While HeartMate II and HeartWare devices still account for a substantial proportion of patients in long‐term follow‐up, our findings—particularly regarding thromboembolic and hemorrhagic events—may not be fully generalizable to patients supported by newer‐generation LVADs, and caution is warranted when applying these results to contemporary clinical practice. Similarly, this time frame preceded the widespread adoption of sodium‐glucose co‐transporter 2 inhibitors, which are now considered foundational therapy in HF management and have been associated with reductions in both AF burden and sudden cardiac death in patients without LVAD support—an area that warrants further investigation in the LVAD population. Second, the diagnosis of AF relied on standard electrocardiographic documentation, which may have missed paroxysmal or intermittent episodes. As such, the true prevalence of AF in this population may have been underestimated. Extended rhythm monitoring (e.g., Holter or continuous telemetry) might have detected a higher AF burden and allowed a more accurate classification—an aspect that should be addressed in future studies. Furthermore, the absence of serial echocardiographic and functional assessments precluded evaluation of potential subclinical effects of pre‐AF. The relatively short median follow‐up of 9.1 months limits the assessment of truly long‐term outcomes in this cohort and should be acknowledged as a limitation of the study. AF management strategies were not standardized and were left to the discretion of the treating physicians. Additionally, our analysis focused exclusively on patients with AF prior to LVAD implantation; incident AF occurring after surgery was not recorded. The subgroup analysis according to AF subtypes was limited by small sample sizes, which may have resulted in reduced statistical power to detect meaningful differences. A major limitation of our study lies in the absence of data on anticoagulation intensity, specifically INR levels, which considerably undermines the strength of conclusions regarding the apparently neutral association between AF and both stroke and pump thrombosis. Indeed, it is plausible that clinicians may have empirically targeted higher INR ranges in patients with preoperative AF, a practice that could have masked an underlying prothrombotic tendency while simultaneously increasing the risk of bleeding—although the latter was not observed, its interpretation remains challenging without precise anticoagulation data. Similarly, data regarding left atrial appendage closure were not available, which precludes assessment of its potential influence on thromboembolic risk in this cohort. Furthermore, the lack of information on rehospitalizations for HF during follow‐up constitutes a critical omission, as these hospitalizations, often driven by right ventricular failure or persistent congestion, represent important morbidity endpoints with substantial impact on quality of life in the LVAD population. While overall survival did not differ between groups, the possibility that preoperative AF may predispose patients to a higher incidence of HF admissions cannot be excluded. Likewise, data regarding prior ventricular tachycardia ablation were not available, limiting our ability to evaluate its potential impact on arrhythmia‐related outcomes.

## 6 Conlusion

AF is common among LVAD recipients, affecting nearly one in two patients at the time of implantation. Such patients have more comorbidities. However, baseline AF does not appear to affect long‐term survival, bleeding, stroke, or pump thrombosis, although it is associated with an increased risk of late VAs. However, these findings are derived from a historical cohort supported exclusively by older‐generation devices (HeartMate II, HeartWare, Jarvik 2000) and thus may not be fully applicable to current practice—particularly in patients implanted with the HeartMate 3, which offers superior hemocompatibility and a lower risk of thrombosis. Further research is warranted to better identify the subset of patients in whom pre‐AF has a true clinical impact, and to determine the most appropriate management strategies in this context.

## Author Contributions

M.C., V.G., R.M.: made substantial contributions to the conception and design of the work, the acquisition, analysis, and interpretation of data, and drafted the work. V.B., P.G., M.D., P.G., P.M., C.D., E.F.: substantively revised the manuscript. All authors read and approved the final manuscript.

## Conflicts of Interest

The authors declare no conflicts of interest.

## Supporting information


**Figure S1:** Love plot of standardized mean differences for propensity‐score matching between patients with and without AF.
