# Supplementary material for: Preoperative Atrial Fibrillation Does Not Impact Long‐Term Survival and Complications in Left Ventricular Assistance Device Recipients
Source: Artif Organs. 2025 Oct 4;50(1):119–28. doi: 10.1111/aor.70020 (PMC12954468; doi:10.1111/aor.70020)
Supplement: Supplementary file 1 — Figure S1: Love plot of standardized mean differences for propensity‐score matching between patients with and without AF. [file AOR-50-119-s001.docx]

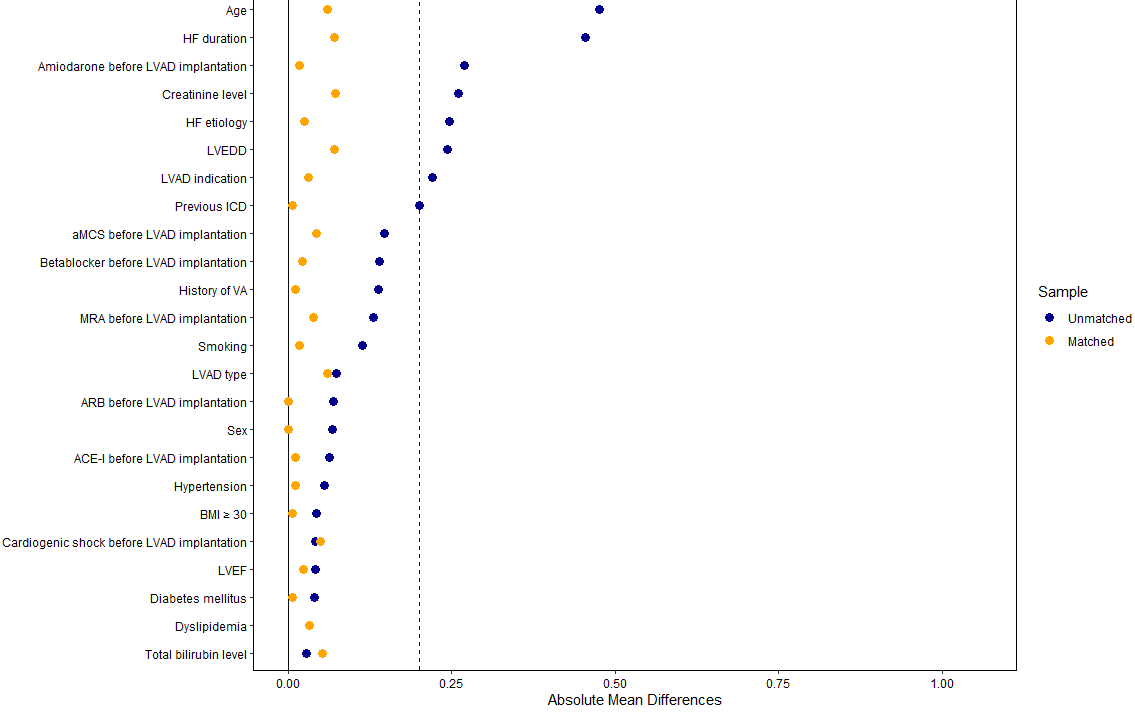


Supplementary Figure 1. Love plot of standardized mean differences for propensity-score matching between patients with and without AF.

ACE-I, angiotensin-converting enzyme; AF, atrial fibrillation; ARB, Angiotensin receptor blockers; BMI, body mass index; LVAD, left ventricular assist device; LVEDD, left ventricular end-diastolic diameter; LVEF, left ventricular ejection fraction; MRA, mineralocorticoid receptor antagonist; VA, ventricular arrhythmia
